# Impact of diagnosis-to-ablation time on left atrial remodeling and voltage-guided ablation outcome in persistent atrial fibrillation patients

**DOI:** 10.3389/fcvm.2026.1751432

**Published:** 2026-03-12

**Authors:** Halim Marzak, François Severac, Clément Baldacini, Simon Fitouchi, Loic Faucher, Julien Jacques, Thomas Cardi, Mohamad Kanso, Alexandre Schatz, Patrick Ohlmann, Olivier Morel, Laurence Jesel

**Affiliations:** 1 Division of Cardiovascular Medicine, Nouvel Hôpital Civil, Strasbourg University Hospital, Strasbourg, France; 2 UR 3074 Translational CardioVascular Medicine, CRBS, University of Strasbourg, Strasbourg, France; 3 Public Health Service, Groupe Méthodes en Recherche Clinique (GMRC), Strasbourg University Hospital, Strasbourg, France

**Keywords:** atrial fibrillation, bipolar voltage map, catheter ablation, diagnosis-to-ablation time, low voltage zones, voltage-guided ablation

## Abstract

**Background:**

Delayed catheter ablation (CA) has been associated with higher rates of atrial fibrillation (AF) recurrence. Low-voltage zones (LVZs) are established predictors of AF recurrence after CA. Data on LVZ assessment in relation to diagnosis-to-ablation time (DAT) remain limited. This study aimed to evaluate the extent of left atrial (LA) LVZs, bipolar voltage, and the outcomes of voltage-guided CA in patients with persistent AF, classified according to DAT.

**Methods:**

We consecutively enrolled 350 patients with persistent AF undergoing their first voltage-guided CA. Patients were classified according to DAT into two groups: DAT ≤1 year (*n* = 131) and >1 year (*n* = 219). LA voltage mapping was performed during sinus rhythm. LVZs were defined as regions with a bipolar voltage <0.5 mV.

**Results:**

Patients with DAT >1 year exhibited lower LA bipolar voltage (*p* < 0.01) and larger LA and indexed LA volumes (*p* < 0.01). LVZs were more frequent in this group (43% vs. 28%, *p* = 0.01), particularly moderate LVZs (*p* = 0.04). Recurrence of atrial tachyarrhythmias (ATs) after a single procedure occurred more often in patients with DAT >1 year (log-rank *p* = 0.05). Multivariable analysis revealed that female sex (*p* < 0.001), indexed LA volume ≥48 mL/m^2^ (*p* = 0.008), age ≥60 years (*p* < 0.05), and P-wave duration ≥150 ms (*p* < 0.001) were independently associated with the presence of LVZs. A history of paroxysmal AF was associated with a lower likelihood of LVZs, whereas DAT was not an independent predictor.

**Conclusion:**

Patients with persistent AF and a longer DAT displayed more extensive LA substrate remodeling. Despite a tailored ablation targeting LVZs, a longer DAT was associated with a higher rate of AT recurrence. Early ablation within the first year after AF diagnosis could optimize AF CA outcomes.

## Introduction

Atrial fibrillation (AF) is the most common cardiac arrhythmia and is associated with an increased risk of cardiovascular death (1), heart failure onset, and stroke (2). Recent data show that early rhythm control (ERC), including treatment with antiarrhythmic drugs (AADs) or/and AF ablation, can reduce cardiovascular outcomes and mortality compared with usual care in patients with AF (3). Furthermore, ERC has been associated with improved quality of life (4).

Importantly, early AF ablation has been associated with a significant reduction in the composite outcome of all-cause death, stroke, and major bleeding compared with AAD therapy (5). Ablation has also been shown to be more effective than AAD therapy in improving both quality of life and symptoms in patients with AF (5) and reducing health care resource utilization and hospitalizations (6). Initial treatment of paroxysmal AF with catheter ablation has also been associated with a lower risk of progression to persistent AF or recurrent atrial tachyarrhythmia compared with first-line AAD therapy (7). The progression of AF results from the continuous interplay of underlying electrical, cellular, and neuro-hormonal mechanisms, combined with clinical parameters and associated cardiovascular risk factors that favor AF and promote atrial substrate remodeling. Risk factors such as age, heart failure, hypertension, diabetes, and chronic kidney disease are known to contribute to this remodeling, which is a key driver of AF progression and is associated with an increased risk of cardiovascular events, hospitalizations, and mortality (8).

Observational studies have shown that the timing of ablation, defined as the diagnosis-to-ablation time (DAT), plays a critical role in outcomes for AF patients. Specifically, a shorter DAT has been independently associated with improved post-ablation outcomes. Early ablation has been linked to significant lower rates of AF recurrence and fewer hospitalizations compared with delayed interventions (9, 10).

Winkle et al. reported that, in patients with persistent AF, early ablation was associated with better outcomes compared with delayed ablation. However, among patients with paroxysmal AF, early ablation did not offer a significant advantage as long as the arrhythmia remained paroxysmal (11).

These recent findings are poised to reshape the future conceptual framework and clinical practice of rhythm management in AF. Despite this, data on left atrial (LA) remodeling in relation to DAT in persistent AF remain limited. One study reported that a longer DAT in patients with persistent AF was correlated with greater LA enlargement (10). Low-voltage zones (LVZs), which reflect structural remodeling of the LA, are recognized as strong predictors of AF recurrence following pulmonary vein isolation (PVI) alone (12). PVI in combination with LVZ-guided ablation may represent an interesting strategy to improve outcomes in patients undergoing ablation for persistent AF. The purpose of our study was to evaluate the extent of LA LVZs and bipolar voltage amplitudes in a cohort of patients with persistent AF, comparing those with a DAT ≤1 year to those with a DAT >1 year.

We also assessed 36-month outcomes following LA voltage-guided ablation based on the timing of ablation and analyzed predictive of the presence of LVZs and atrial tachyarrhythmia (AT) recurrence after catheter ablation (CA) in the entire cohort.

## Methods

### Study population

Among 574 patients referred to our institution (Strasbourg University Hospital, France) from November 2019 to February 2023 for a first radiofrequency ablation of persistent AF, 350 (61%) patients were included in the study. Patients with structural heart disease (88 patients) were excluded, as were 136 patients without an LA voltage map in sinus rhythm (SR). Only patients with persistent AF at the time of the index procedure were included; those referred for catheter ablation of paroxysmal AF or for redo procedures were excluded. At our center, ablation is performed as early as possible to minimize atrial remodeling and disease progression.

Due to the retrospective design, detailed longitudinal data on antiarrhythmic drug exposure, cardioversion frequency, and structured risk-factor management between atrial fibrillation diagnosis and ablation were not consistently available and were therefore excluded from the analysis.

A history of paroxysmal AF was defined as a documented episode of paroxysmal AF prior to the onset of persistent AF, based on the medical records of patients. This variable does not reflect AF burden or the number of paroxysmal episodes.

**Figure 1:**
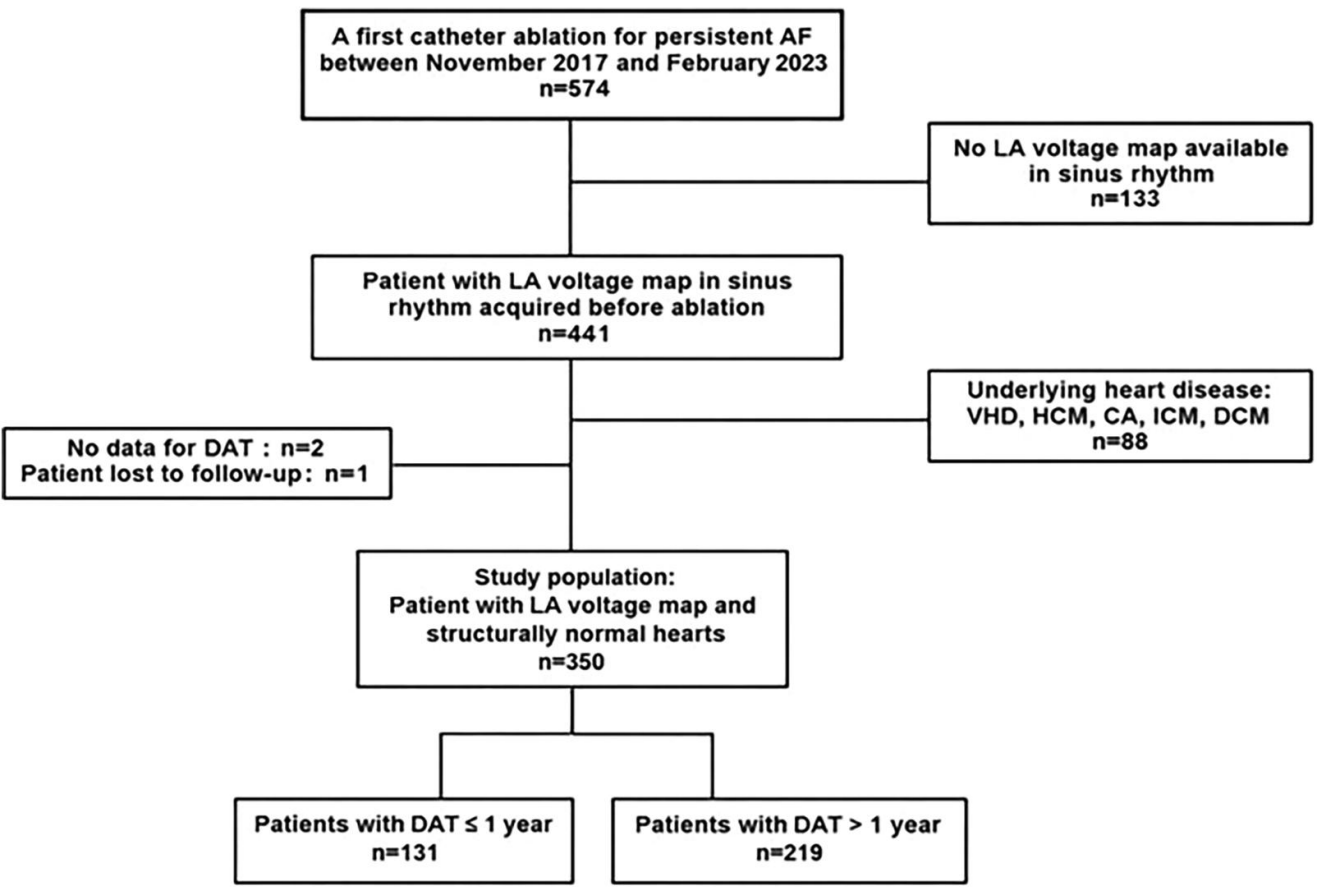
Flowchart of the study. Among 574 patients admitted for initial radiofrequency ablation of persistent AF between November 2019 and February 2023, 350 (61%) patients met the inclusion criteria. A total of 133 patients (23%) had no LA voltage mapping in sinus rhythm, and 91 (16%) were excluded from the analysis: 88 due to structural heart disease, two because of missing DAT data, and one due to loss to follow-up. AF, atrial fibrillation; LA, left atrial; VHD, valvular heart disease; HCM, hypertrophic cardiomyopathy; CA, cardiac amyloidosis; ICM, ischemic cardiomyopathy; DCM, dilated cardiomyopathy; DAT, diagnosis-to-oblation time.

Structural heart disease was defined as the presence of ischemic heart disease, valve dysfunction (≥moderate), or primary myocardial structural disease, including dilated cardiomyopathy and hypertrophic cardiomyopathy (Figure 1).

DAT was defined as the interval between the first documented episode of AF and the day of CA. The unmatched cohort was then divided into two groups: DAT ≤1 year (*n* = 131) and DAT >1 year (*n* = 219). Patient demographics and baseline clinical characteristics are summarized in Table 1.

**Table 1:** Baseline characteristics according to DAT.

Variable	DAT ≤ 1 year (*n* = 131)	DAT > 1 year (*n* = 219)	*P*-value
Age, years	64 [56–70]	67 [61–72]	**0**.**01**
Female gender	32 (24)	72 (33)	0.11
BMI, kg/m^2^	29 [26–34]	30 [26–33]	0.78
Dyslipidemia, *n* (%)	52 (40)	95 (43)	0.53
Hypertension, *n* (%)	86 (66)	145 (66)	0.93
Diabetes mellitus, *n* (%)	28 (21)	35 (16)	0.27
Smoking, *n* (%)	20 (15)	21 (10)	0.16
OSA, *n* (%)	32 (24)	68 (31)	0.21
CHA_2_DS_2_-VASc score	2 (1–3)	2 (1–3)	0.76
Paroxysmal AF history, *n* (%)	16 (12)	129 (59)	**<0**.**01**
Sinus node dysfunction, *n* (%)	10 (8)	19 (9)	0.87
Time to treatment, days	186 [133–251]	1,286 [665–2,366]	**<0**.**01**
Reported AF duration, months			**<0**.**01**
<3 months	100 (76)	132 (60)	
≥3 to <6 months	13 (10)	32 (15)	
≥6 to <9 months	9 (7)	24 (11)	
≥9 to <12 months	9 (7)	7 (3)	
≥12 months	0 (0)	24 (11)	
eGFR, mL/min/1.73 m^2^	83 [66–93]	82 [67–93]	0.5
LVEF, %	60 [54–65]	60 [55–65]	0.17
P-wave duration, ms	141 ± 22	150 ± 25	**<0**.**01**
Beta-blocker	109 (83)	173 (79)	0.5
ACEi/ARB	66 (50)	120 (55)	0.45
Aldosterone receptor antagonist	21 (16)	32 (15)	0.86
Antiarrhythmic drugs prior to the procedure	124 (95)	195 (89)	0.11
Amiodarone	94 (76)	146 (75)	0.92
Flecainide	29 (23)	43 (22)	0.92
Sotalol	1 (1)	6 (3)	0.25

Data are presented as values (with percentages) for categorical variables and as median (25th–75th percentile) for quantitative variables. A two-tailed *p*-value < 0.05 was considered significant. Bold values indicate statistical significance at *p* < 0.05.

DAT, diagnosis-to-ablation time; BMI, body mass index; OSA, obstructive sleep apnea; AF, atrial fibrillation; ACEi/ARB, angiotensin-converting enzyme inhibitor/angiotensin II receptor blocker; eGFR, estimated glomerular filtration rate; LVEF, left ventricular ejection fraction; LAA, left atrial appendage.

Diagnosis-to-ablation time = time from the first clinical diagnosis of AF to the ablation procedure.

The study protocol was approved by the institutional review board of Strasbourg University (CE-2023-113). Written informed consent for the ablation procedure and for the use of data for research purposes was obtained from all patients during the consultation before the CA procedure.

### Electro-anatomical voltage mapping

All CA procedures were performed under general anesthesia. Cardiac computed tomography (CT) was performed prior to each procedure to visualize LA anatomy. Left atrial volume (LAV), excluding the left atrial appendage (LAA), was measured from the CT scan for each patient and expressed in milliliters (mL). The left atrial volume index (LAVI) corresponded to the LAV indexed to body surface area and was expressed in mL/m^2^.

The electro-anatomical mapping system (CARTO 3, Biosense Webster, Diamond Bar, CA, USA) was used for mapping and ablation guidance. LA endocardial mapping was performed using a multielectrode mapping catheter (PentaRay®, Biosense Webster, Diamond Bar, CA, USA) to create a high-density bipolar LA endocardial voltage map in SR at the beginning of the procedure. For patients with AF, electrical conversion to SR was performed before the creation of the LA map. The quality of all acquired voltage points was reviewed for each patient. A stable catheter contact for at least two consecutive beats validated the acquisition of points recorded during LA mapping. Mechanically induced premature beats were excluded after verification of all points recorded in SR.

The LA was segmented into six anatomical regions: anterior, septal, posterior, lateral, inferior, and LAA. The roof was part of the anterior region, as previously described (13). Bipolar voltage amplitude was automatically recorded at each mapping point during LA mapping to calculate the global LA bipolar voltage amplitude, which was defined as the mean of all voltage amplitudes of each point recorded during LA mapping.

LVZs were defined as contiguous areas of at least 1 cm^2^ showing a bipolar peak-to-peak electrogram voltage of <0.5 mV in patients who were in SR at the time of ablation (14).

The degree of LVZ extent in the LA was determined semi-quantitatively for each patient and categorized according to the UTAH fibrosis classification (15) as follows: stage I (no or discret LVZ, ≤5%), stage II (mild, >5 to ≤20%), stage III (moderate, >20 to ≤35%), and stage IV (severe, >35%). Patients with LA LVZ ≥5% were classified as having LVZ, whereas those with LA LVZ <5% were considered non-LVZ. Any atrial region containing LVZ was defined as a low-voltage region.

Left atrial intracavitary volume (LAIV), excluding the LAA, was measured for each patient after LA anatomic reconstruction and expressed in mL. The left atrial intracavitary volume index (LAIVI) corresponded to the LAIV indexed to the body surface area and was expressed in mL/m^2^.

### Catheter ablation procedure

All patients underwent antral PVI during the CA procedure according to the CLOSE protocol (16). Radiofrequency (RF) applications were delivered using a 4-mm irrigated contact-force ablation catheter (ThermoCool® SmartTouch®, Biosense Webster, Diamond Bar, CA, USA). PVI was performed using a high-power, short-duration ablation strategy by applying 50 and 40 W to the anterior and posterior segments of pulmonary veins, respectively (17). RF energy was delivered until achieving an ablation index (AI) of 450–500 at the anterior wall and 350–400 at the posterior wall. A contact force of 5–20 g was obtained at each site, with a lesion tag size of 2 mm, an interlesion distance of <6 mm, a target temperature of 43°C, and an infusion rate of 17 mL/mn. PVI was only performed in patients with a normal LA voltage map (stage I). For the other patients (stages II–IV), an additional ablation targeting the LVZ was performed by isolation or homogenization. Linear ablation across LVZs was achieved when the LVZ ablation area could be considered a critical isthmus site for potential macro-reentrant tachycardia. Linear lesions were also created to isolate large LVZ areas from the rest of the left atrial tissue, such as constructing a posterior box using a roof line and an inferoposterior line. At the end of the procedure, atrial burst pacing from the coronary sinus (CS) was performed to induce any tachycardia. No additional ablation was performed in the case of induced AF.

### Follow-up

AADs were continued in all patients during a 3-month blanking period of after CA. Arrhythmic episodes occurring within the ﬁrst 3 months were excluded from the analysis of ﬁnal success rates. Patients were followed regularly by their cardiologist at 3 months and every 6 months thereafter, up to 42 months after RF CA. At each outpatient visit, patients were asked to give details of their symptoms. A 12-lead electrocardiogram (ECG) and 24-h ambulatory Holter monitoring were also performed.

For this study cohort, AT recurrence was defined as any AF, atrial flutter, or atrial tachycardia lasting more than 30 s recorded on a routine or symptom-triggered ECG during an outpatient visit after a 3-month blanking period. AADs were gradually discontinued between 3 and 6 months after ablation at the physician's discretion, provided no arrhythmia recurrence had occurred.

### Statistical analysis

Categorical variables are reported as counts with percentages, and continuous variables are expressed as medians with interquartile ranges. An inverse probability of treatment weighting (IPTW) approach was used to balance the baseline characteristics between the two groups (early or late ablation). A propensity score was estimated using a multivariable logistic regression model. The dependent variable was the timing of ablation, and the independent variables included all potential confounders of the exposure–outcome relationship [AF duration, age, coronary artery disease, gender, smoking status, obstructive sleep apnea (OSA), diabetes mellitus, dyslipidemia, body mass index, estimated glomerular filtration rate, CHA_2_DS_2_-VASc score, hypertension, and history of paroxysmal AF]. Stabilized weights were then computed to generate a pseudopopulation (weighted cohort) in which the distribution of baseline covariates was independent of the timing of ablation. We used absolute standardized differences (ASD) to assess the comparability of the baseline covariates between the two groups. Covariates with an ASD <0.1 indicate non-meaningful imbalance (Supplementary Figure 1) (18).

For each binary outcome, we performed univariable weighted logistic regression to estimate the odds ratio (OR) for the late ablation group and its 95% confidence interval (CI). For continuous variables, differences were estimated using linear regression models. Normality of residuals and homogeneity of variance were checked graphically.

Factors associated with the presence of LVZs were identified using a multivariable logistic regression model. We included all variables with clinical relevance. Factors related to AT recurrence were identified using a multivariable Cox model. Results are presented as hazard ratios (HRs) with 95% CIs.

Kaplan–Meier survival curves were constructed for each group to analyze freedom from AT recurrence after a single procedure. The two groups were compared using the log-rank test with a Bonferroni correction.

To avoid potential bias arising from dichotomizing DAT at a 1-year cutoff in the analysis of its relationship with LVZs, sensitivity analyses were performed treating DAT as a continuous variable (OR per 30-day increase) and introducing restricted cubic splines (RCS) with three knots at the 10th, 50th, and 90th percentiles in the multivariable model to assess a potential non-linear effect.

A two-tailed *p-*value of <0.05 was considered statistically significant. All analyses were performed using R software (R Core Team, 2021; R Foundation for Statistical Computing, Vienna, Austria).

## Results

### Baseline characteristics

Patient characteristics of the primary cohort before and after IPTW are summarized in Tables 1, 2. Before weighting, the traditional cardiovascular risk factors were equally distributed in the two groups. Patients in the DAT >1 year group were older [67 (61–72) vs. 64 (56–70) years, *p* = 0.01] and had a longer duration of AF. They also had a higher prevalence of a history of paroxysmal AF (59% vs. 12%, *p* < 0.01). The higher prevalence of a history of paroxysmal AF in the DAT >1 year group likely reflects the natural progression from paroxysmal to persistent AF over time, consistent with the longer overall AF duration and older age observed in this group. No differences were observed for the other variables between the two groups (Table 1). After weighting, no differences were observed concerning these variables between the two groups (Table 2).

**Table 2:** Baseline characteristics according to DAT before and after IPTW.

Variable	Before IPTW			After IPTW		
	DAT ≤ 1 year (*n* = 131)	DAT > 1 year (*n* = 219)	ASD	DAT ≤ 1 year (*n* = 129.7)	DAT > 1 year (*n* = 218.7)	ASD
Age, years	63 [56–70]	67 [61–72]	**0**.**307**	68 [56–71]	66 [59–71]	0.052
Female gender	32 (24%)	72 (33%)	**0**.**188**	40 (31%)	67 (30%)	0.016
AF duration			**0**.**376**			0.096
<3 months	101 (77%)	132 (60%)		91 (70%)	145 (66%)	
≥3 to <6 months	13 (10%)	32 (15%)		17 (13%)	29 (13%)	
≥6 months	17 (13%)	55 (25%)		22 (17%)	45 (21%)	
BMI, kg/m^2^	29 [26–34]	30 [26–33]	0.086	28 [27–31]	30 [26–33]	0.097
CHA_2_DS_2_-VASc score	2 [1–3]	2 [1–3]	0.057	2 [1–4]	2 [1–3]	0.018
Dyslipidemia	51 (39%)	95 (43%)	0.09	62 (48%)	95 (44%)	0.085
Hypertension	86 (66%)	145 (66%)	0.012	91 (71%)	147 (67%)	0.076
Diabetes mellitus	28 (21%)	35 (16%)	**0**.**139**	23 (18%)	40 (18%)	0.012
Smoking	20 (15%)	21 (10%)	**0**.**173**	18 (14%)	25 (11%)	0.075
OSA	32 (24%)	68 (31%)	**0**.**148**	34 (26%)	61 (28%)	0.048
Coronary artery disease	25 (19%)	24 (11%)	**0**.**229**	19 (15%)	31 (14%)	0.012
eGFR, mL/min/1.73 m^2^	83 [66–94]	82 [67–93]	0.071	86 [66–94]	81 [67–93]	0.092
Paroxysmal AF history	16 (12%)	129 (59%)	**1**.**117**	53 (41%)	91 (41%)	0.017

Data are presented as values (with percentages) for categorical variables and as median (25th–75th percentile) for quantitative variables. Bold values indicate statistical significance with ASD ≥ 0.1.

ASD values close to 0 indicate insignificant differences between groups. We consider that covariates with ASD <0.1 indicate an unmeaningful imbalance.

IPTW, inverse probability of treatment weighting; ASD, absolute standardized differences; DAT, diagnosis-to-ablation time; BMI, body mass index; OSA, AF, atrial fibrillation; eGFR, estimated glomerular filtration rate.

### Differences in left atrial remodeling in the unweighted cohort

Patients with DAT >1 year had greater indexed (72 ± 19 vs. 65 ± 18 mL/m^2^, *p* < 0.01) or non-indexed (150 ± 39 vs. 137 ± 35 mL, *p* < 0.01) LA volumes on CT scan (Table 3). The global LA bipolar voltage amplitude was significantly lower in patients with DAT >1 year in the unweighted cohort [1.8 [1.2–2.5] vs. 2.3 [1.5–2.7] mV, *p* < 0.01] (Table 3). LVZs were more frequently observed in patients with DAT >1 year [93 [43%] vs. 37 [28%], *p* = 0.01] (Table 3). LVZ areas ranging from 20% to 35% of LA (*p* = 0.04), anterior (*p* = 0.03), and posterior (*p* = 0.03) localizations were more frequently observed in patients with DAT >1 year (Table 3). In this unweighted cohort, P-wave duration (PWD) was longer (150 ± 25 vs. 141 ± 22 ms, *p* < 0.01) in the DAT >1 year group (Table 3).

**Table 3:** Analysis of left atrial remodeling parameters between DAT  ≤ 1 year and DAT  > 1 year.

Variable	DAT ≤ 1 year (*n* = 131)	DAT > 1 year (*n* = 219)	*P*-value
LAV on CT scan, mL	137 ± 35	150 ± 39	**<0**.**01**
LAVI on CT scan, mL/m^2^	65 ± 18	72 ± 19	**<0**.**01**
Per-procedural LAIV excluding LAA, mL	132 ± 31	143 ± 37	**0**.**01**
Per-procedural LAIVI excluding LAA, mL/m^2^	63 ± 16	69 ± 17	**<0**.**01**
LA bipolar voltage	2.3 [1.5–2.7]	1.8 [1.2–2.5]	**<0**.**01**
LVZs, *n* (%)	37 (28)	93 (43)	**0**.**01**
LVZ extent in LA			
Mild LVZs, *n* (%)	25 (19)	54 (25)	0.26
Moderate LVZs, *n* (%)	6 (5)	25 (11)	**0**.**04**
Severe LVZs, *n* (%)	6 (5)	14 (6)	0.62
Number of regional LVZs			
Anterior, *n* (%)	34 (26)	82 (38)	**0**.**03**
Septal, *n* (%)	22 (17)	49 (22)	0.24
Posterior, *n* (%)	11 (8)	37 (17)	**0**.**03**
Inferior, *n* (%)	2 (2)	11 (5)	0.14
Lateral, *n* (%)	1 (1)	4 (2)	0.65
LAA, *n* (%)	5 (4)	13 (6)	0.52

All data are presented as values (percentages) for categorical variables or as median (25th–75th percentile) for quantitative variables. A two-tailed *p*-value < 0.05 was considered significant. Bold values indicate statistical significance at *p* < 0.05.

DAT, diagnosis-to-ablation time; LAV, left atrial volume; LAVI, left atrial volume index; CT, computed tomography; LAIV, left atrial intracavitary volume; LAIVI, left atrial intracavitary volume index; LA, left atrium; LVZ, low-voltage zone; LAA, left atrial appendage.

### Differences in left atrial remodeling in the weighted cohort

After IPTW, non-indexed LA volume on CT scan remained higher in patients with a DAT >1 year (*p* = 0.031), while indexed values did not differ significantly (Table 4). LVZs were more frequent in patients with DAT >1 year, although this did not reach significance [93 [43%] vs. 38 [30%], *p* = 0.076]. Posterior LVZs were more frequent in late ablation (17.3% vs. 5.8%, *p* = 0.003), but overall LVZ burden and regional distribution were similar between the two groups (Table 4). PWD remained significantly longer in patients with a DAT >1 year (149 ± 26 vs. 142 ± 22 ms, *p* = 0.031) (Table 4).

**Table 4:** Extent and distribution of low-voltage zones (LVZs) according to DAT after IPTW.

Variable	DAT ≤ 1 year (*n* = 129.7)	DAT > 1 year (*n* = 218.7)	OR [95% CI]	*P*-value
Low-voltage zone extent				
LVZs, *n* (%)	38.3 (29.6)	92.8 (42.6)	1.77 [0.94–3.30]	0.076
Mild LVZs, *n* (%)	37.9 (29.2)	92.8 (42.6)	1.80 [0.96–3.37]	0.069
Moderate LVZs, *n* (%)	12.8 (9.9)	37.8 (17.3)	1.91 [0.74–4.93]	0.180
Severe LVZs, *n* (%)	7.9 (6.1)	14.4 (6.6)	1.09 [0.27–4.41]	0.907
Low-voltage zone distribution				
Anterior LVZs, *n* (%)	36.2 (27.9)	79.1 (36.3)	1.47 [0.77–2.80]	0.244
Septal LVZs, *n* (%)	20.6 (15.9)	52.5 (24.1)	1.68 [0.80–3.50]	0.171
Posterior LVZs, *n* (%)	7.5 (5.8)	37.7 (17.3)	3.39 [1.51–7.59]	0.003
Inferior LVZs, *n* (%)	5.9 (4.5)	14.4 (6.6)	1.49 [0.25–8.88]	0.658
Lateral LVZs, *n* (%)	0.6 (0.4)	5.2 (2.4)	5.55 [0.55–56.07]	0.147
LAA LVZs, *n* (%)	3.5 (2.7)	10.1 (4.6)	1.77 [0.59–5.33]	0.312
≥3 low-voltage regions, *n* (%)	14.7 (1.3)	27.9 (12.8)	1.15 [0.45–2.94]	0.770
Left atrial volume and its index				
LAV on CT scan	139.5 ± 32.6	150.0 ± 40.4	10.5 [0.96–20.1]	**0**.**031**
LAVI on CT scan	67.7 ± 18.0	71.5 ± 18.9	3.89 [−1.34–9.13]	0.145
Per-procedural LAIV, mL	135.6 ± 29.8	143.0 ± 37.9	7.39 [−1.32–16.1]	0.096
Per-procedural LAIVI, mL/m^2^	65.6 ± 16.4	68.1 ± 17.3	2.55 [−2.1–7.2]	0.281
PWD, ms	141.8 ± 22.2	149.5 ± 25.7	7.68 [0.69–14.67]	**0**.**031**
PWD ≥ 150 ms, *n* (%)	32.8 (25.5)	96.2 (44.7)	2.36 [1.28–4.37]	**0**.**006**

All data are presented as values (percentages) for categorical variables or as mean ± SD for quantitative variables. A two-tailed *p*-value < 0.05 was considered significant. Bold values indicate statistical significance at *p* < 0.05.

IPTW, inverse probability of treatment weighting; LVZ, low-voltage zone; OR, odds ratio; CI, confidence interval; DAT, diagnosis-to-ablation time; LAA, left atrial appendage; LA, left atrium; LAV, left atrial volume; LAVI, left atrial volume index; CT, computer tomography; LAIV, left atrial intracavitary volume; LAIVI, left atrial intracavitary volume index; PWD, P-wave duration.

### Outcomes after catheter ablation in the unweighted cohort

The median number of total mapping points collected per map was similar between these two groups [779 (550–1,093) vs. 729 (512–1,078), *p* = 0.82] (Table 5).

**Table 5:** Procedural data-related characteristics.

Variable	DAT ≤ 1 year (*n* = 131)	DAT > 1 year (*n* = 219)	*P*-value
Fluoroscopic time, min	25 [18–31]	22 [17–30]	0.35
Procedure time, min	127 [110–150]	126 [106–147]	0.62
Median total mapping points per patient	729 [512–1,078]	779 [550–1,093]	0.82
Only PVI, *n* (%)	94 (72)	125 (57)	**0**.**01**
LVZ ablation, *n* (%)	37 (28)	93 (43)	**0**.**01**
Linear ablation, *n* (%)	14 (38)	57 (62)	0.03
Anterior line, *n* (%)	8 (57)	27 (47)	0.72
Posterior line, *n* (%)	10 (71)	32 (56)	0.46
Roof line, *n* (%)	8 (57)	39 (68)	0.53
Septal line, *n* (%)	3 (21)	4 (7)	0.13
Mitral line, *n* (%)	5 (36)	8 (14)	0.12
Complications, *n* (%)	7 (5)	7 (3)	0.49
Scarpa's hematoma, *n* (%)	5 (4)	3 (1)	0.16
Stroke, *n* (%)	0 (0)	1 (0.5)	1
Cardiac tamponade, *n* (%)	0 (0)	2 (0.9)	0.6
Right phrenic paralysis, *n* (%)	1 (0.8)	1 (0.5)	1

Data are presented as values (with percentages) for categorical variables or as median (25th–75th percentile) for quantitative variables. A two-tailed *p* value of <0.05 was considered significant. Bold values indicate statistical significance at *p* < 0.05.

DAT, diagnosis-to-ablation time; PVI, pulmonary vein isolation; LVZ, low-voltage zone.

PVI was successfully performed in all patients. PVI alone was performed more frequently in patients with DAT ≤1 year [94 [72%] vs. 125 [57%], *p* = 0.01] compared with those with DAT >1 year (Table 5). After applying IPTW, the results remained similar but did not achieve statistical significance (Supplementary Table 1).

Among all patients with LVZ ablation, no difference in linear ablation was observed between the two groups. There was no difference in procedure time (*p* = 0.62) or fluoroscopy time (*p* = 0.35). Procedure-related complications did not differ between the two groups (Table 5).

**Figure 2:**
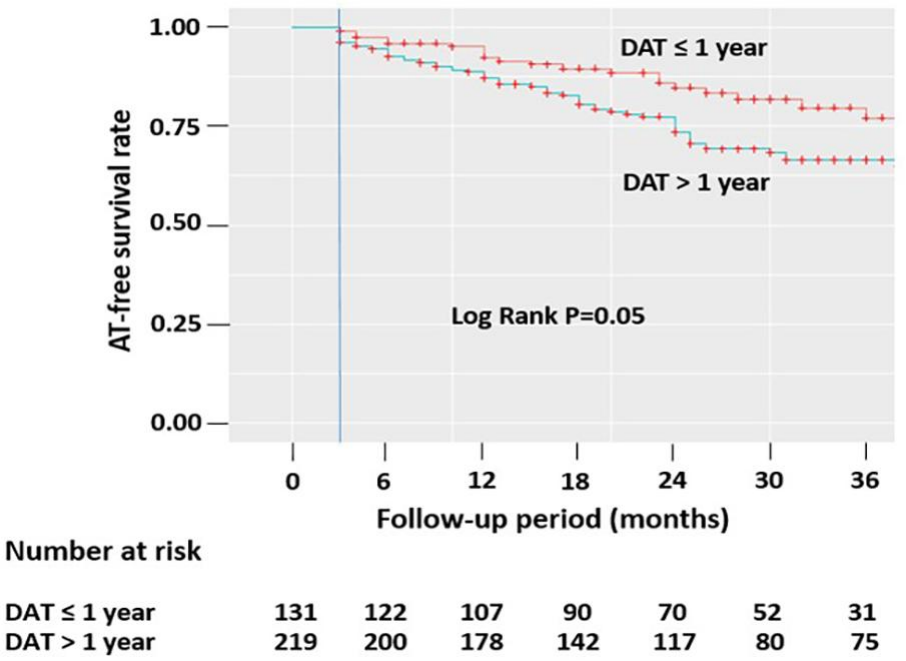
Kaplan–Meier survival curves showing the cumulative AF/AT recurrence-free survival rates according to DAT after a single procedure. ATs, atrial tachyarrhythmias; DAT, diagnosis-to-ablation time; AF, atrial fibrillation; AT, atrial tachycardia.

In the unweighted cohort, AT recurrence was observed in 86 of 350 (24.6%) patients after a single procedure, with a median follow-up of 49 (46–53) months. We observed a significant difference in AT-free survival after a single procedure between the two groups, with a higher recurrence rate in patients with DAT >1 year (log-rank test, *P* = 0.05). After 36 months, 77% of patients with DAT ≤1 year and 67% of those with DAT >1 year remained free from ATs (Figure 2). Kaplan–Meier survival curves are shown in Figure 2.

In the whole cohort, AADs were discontinued in 75% (263/350) of the patients. AAD discontinuation was more frequent in patients with DAT ≤1 year [110 [84%] vs. 153 [70%], *p* < 0.01] compared with those with DAT >1 year.

### Predictors of low-voltage zones

To identify predictors of LVZs, univariable and multivariable analyses were performed in the whole original population. Multivariable analysis showed that female sex (*p* < 0.001), indexed LA volume ≥48 mL/m^2^ (*p* = 0.008), age 60–75 years (*p* = 0.008), age above 75 years (*p* = 0.033), and PWD ≥150 ms (*p* < 0.001) was independently associated with the presence of LVZs (Table 6). A history of paroxysmal AF was independently associated with a lower likelihood of LVZs (OR 0.41, 95% CI 0.20–0.86, *p* = 0.019), whereas DAT was not a significant predictor (Table 6).

**Table 6:** Predictors of the presence of low-voltage zones in the global population.

Variable	Univariable analysis			Multivariable analysis		
	OR	95% CI	*P* Value	OR	95% CI	*P*-value
DAT > 1 year	1.89	1.19–3.01	0.007	1.53	0.72–3.26	0.272
Age 60–75 years	7.00	3.35–14.64	<0.001	4.45	1.69–11.75	**0**.**003**
Age >75 years	23.76	8.92–63.25	<0.001	6.36	1.61–25.05	**0**.**008**
Female gender	5.58	3.39–9.16	<0.001	4.80	2.39–9.67	**<0**.**001**
Hypertension	2.76	1.67–4.56	<0.001	1.50	0.68–3.32	0.317
Diabetes mellitus	1.56	0.90–2.71	0.113	1.81	0.77–4.28	0.174
OSA	0.93	0.57–1.50	0.760			
BMI	0.90	0.59–1.39	0.647			
CHA_2_DS_2_-VASc score ≥2	6.12	3.30–11.32	<0.001	1.76	0.66–4.68	0.256
eGFR <60 mL/min/1.73 m^2^	3.34	1.84–6.06	<0.001	0.98	0.41–2.32	0.964
AF duration >6 months	1.30	0.77–2.21	0.329			
LAVI on CT ≥48 mL/m^2^	11.96	2.82–50.67	0.001	11.55	2.07–64.57	**0**.**005**
PWD ≥ 150 ms	12.18	7.21–20.57	<0.001	10.44	5.42–20.14	**<0**.**001**
Paroxysmal AF history	1.60	1.03–2.49	0.036	0.41	0.20–0.86	**0**.**019**

Data are presented as odds ratios with 95% CIs. A two-tailed *p*-value < 0.05 was considered significant. Bold values indicate statistical significance at *p* < 0.05.

OR, odds ratio; CI, confidence interval; DAT, diagnosis-to-ablation time; OSA, obstructive sleep apnea; BMI, body mass index; eGFR, estimated glomerular filtration rate; AF, atrial fibrillation; LAVI, left atrial volume index; CT, computed tomography; PWD, P-wave duration.

Diagnosis-to-ablation time = time from the first clinical diagnosis of AF to the ablation procedure.

To assess the robustness of our findings, a sensitivity analysis was performed considering DAT as a continuous variable (OR reported per 30-day increase). DAT was not significantly associated with the presence of LVZs [OR 1.00 (0.99–1.01), *p* = 0.697] (Supplementary Table 2). In addition, a sensitivity analysis using restricted cubic splines was conducted. The overall association between DAT and the presence of LVZs was not significant (*p*-overall = 0.547), and there was no evidence of non-linearity (*p*-non-linear = 0.306) (Supplementary Figure 2).

### Predictive factors of AT recurrence

To identify predictors of AT recurrence after CA, univariable and multivariable analyses were performed in the whole original cohort. No predictive factor of AT recurrence after CA could be identified (Table 7).

**Table 7:** Predictors of AT recurrence after catheter ablation in the global population.

Variable	Univariable analysis			Multivariable analysis		
	HR	95% CI	*P*-value	HR	95% CI	*P* value
DAT >1 year	1.59	0.99–2.55	0.053	1.26	0.73–2.17	0.401
Age 60–75 years	0.76	0.47–1.22	0.256			
Age >75 years	0.93	0.45–1.92	0.834			
Female gender	1.23	0.79–1.92	0.368			
Hypertension	0.77	0.50–1.19	0.237			
Diabetes mellitus	0.64	0.34–1.20	0.162	0.61	0.31–1.20	0.151
OSA	1.33	0.85–2.07	0.212			
BMI	0.95	0.62–1.45	0.818			
CHA_2_DS_2_-VASc score ≥2	0.88	0.56–1.36	0.554			
eGFR <60 mL/min/1.73 m^2^	0.99	0.54–1.83	0.977			
AF duration >6 months	1.42	0.85–2.37	0.185	1.27	0.73–2.22	0.392
LAVI on CT ≥48 mL/m^2^	1.20	0.55–2.60	0.646	0.88	0.39–1.97	0.750
LVZ presence	1.51	0.98–2.31	0.061	1.27	0.75–2.16	0.373
PWD ≥150 ms	1.38	0.90–2.12	0.141	1.17	0.69–1.99	0.555
Paroxysmal AF history	1.53	1.00–2.33	0.050	1.21	0.74–1.98	0.452

Data are presented as odds ratios with 95% CIs. A two-tailed *p*-value < 0.05 was considered significant. Bold values indicate statistical significance at *p* < 0.05.

ATs, atrial tachyarrhythmias; HR, hazard ratio; CI, confidence interval; DAT, diagnosis-to-ablation time; OSA, obstructive sleep apnea; BMI, body mass index; eGFR, estimated glomerular filtration rate; AF, atrial fibrillation; LAVI, left atrial volume index; LVZ, low-voltage zone; CT, computed tomography.

Diagnosis-to-ablation time = time from the first clinical diagnosis of AF to the ablation procedure.

## Discussion

### Main findings

In the present study, we report that, in a cohort of patients with persistent AF, those with DAT >1 year exhibited more advanced LA electrophysiological remodeling, characterized by lower bipolar voltage, a greater extent of LVZ, and increased LA volume, compared with those with DAT ≤1 year.

During a 36-month follow-up after a single voltage-guided ablation, patients with a longer DAT experienced a higher rate of AT recurrence. AF ablation consisted of PVI alone in 72% of patients with a shorter DAT, compared with 57% in patients with a longer DAT. Female sex, indexed LA volume ≥48 mL/m^2^, age ≥60 years, and PWD ≥150 ms were identified as independent predictors of LVZ across the cohort. In contrast, a history of paroxysmal AF was independently associated with a lower likelihood of LVZs, whereas DAT was not an independent predictor after adjustment.

However, no independent predictor of AT recurrence could be identified in this analysis.

### Differences in baseline characteristics

We observed several baseline differences between the two groups. Unsurprisingly, patients with a longer DAT tended to be older. In clinical practice, CA is typically not considered a first-line strategy in elderly patients, which may lead to delayed intervention. In addition, these patients exhibited a longer duration of AF prior to ablation and a higher prevalence of a history of paroxysmal AF at baseline. The higher rate of prior paroxysmal AF in patients with a longer DAT likely reflects the natural progression from paroxysmal to persistent AF over time, consistent with the longer overall AF duration and older age observed in this group of patients. Since these patients are older and have more limited access to ablation, the delay is therefore longer.

Previous studies have reported a natural progression from paroxysmal to persistent and long-standing persistent AF after the initial diagnosis, which reflects the chronic and evolving nature of the disease (19). Indeed, AF itself promotes electrical, cellular, and structural changes in the atria, contributing to the development of pro-arrhythmic LA substrate remodeling, establishing a self-perpetuating cycle commonly referred to as “AF begets AF.”

### Differences in left atrial remodeling according to DAT

Data on LA electrophysiologic remodeling, bipolar voltage, and LVZs in patients with persistent AF stratified by DAT remain scarce. Our study is the first to provide a detailed analysis of LA voltage mapping in patients with DAT >1 year, demonstrating significantly lower LA bipolar voltage compared with those with DAT ≤1 year.

While several studies have reported an association between increased LA size and longer DAT, none have provided detailed assessments of voltage amplitude or the presence of LVZs (9, 10). Hussein et al. were the only ones to report a significant increase in LA surface area (*P* = 0.03) with a longer DAT (10). In contrast, other studies did not observe differences in LA diameter based on DAT (9). However, LA diameter measurement is no longer recommended by echocardiographic societies due to its low reproducibility and tendency to underestimate LA enlargement (20).

In our study, we highlight that LA volume, measured by CT scanning, remained significantly higher in patients with DAT >1 year, even after adjustment using IPTW, compared with those with a shorter DAT. These findings provide further evidence of a strong association between DAT and the extent of atrial remodeling, as reflected by structural atrial dilatation. Atrial remodeling encompasses both structural changes, such as atrial dilation, and electrophysiological alterations, including the extent of fibrosis. In our study, LVZs were observed in 43% of patients with a longer DAT compared with 28% of those with a shorter DAT. For the first time, we provide detailed information on both the extent and regional distribution of LA LVZs in patients with persistent AF stratified by DAT.

Analysis of the LVZ amount revealed a higher prevalence of moderate LVZs in the longer DAT group. Consistent with prior studies (14), LVZs were most commonly located in the septum and in the anterior and posterior walls in both groups. However, in the longer DAT group, LVZs were significantly more frequent in the anterior and posterior walls.

Our findings are consistent with a prospective, non-randomized cohort study that demonstrated progression of atrial structural remodeling over 12 months in patients with high-burden paroxysmal AF. This progression was characterized by a decline in LA total strain and prolongation of the P-wave. In contrast, early AF ablation was associated with significant reversal of LA remodeling (21). Notably, reduced LA total strain was correlated strongly with advanced LA remodeling, as evidenced by lower LA voltage amplitude, prolonged LA activation time, and a greater prevalence of low-voltage electrograms (21).

In our data, PWD was significantly longer in patients with a longer DAT, even after adjustment using IPTW, consistent with the findings reported by Walters et al. (21). Several factors may influence PWD. While LA dilation can contribute to PWD prolongation, its impact is generally modest (22). Notably, a few studies have highlighted an association between prolonged PWD and a low-voltage LA substrate in patients with both persistent and paroxysmal AF (23). These observations reinforce the association between advanced structural LA remodeling and prolonged PWD in patients with a longer DAT.

### Favoring factors of LVZs

We identified several predictive factors of LVZs in the overall cohort, including advanced age, female gender, indexed LA volume, and PWD ≥150 ms. The first three variables are well-established predictors of LVZs in patients with AF. Previous studies have consistently demonstrated a correlation between increasing age and the presence of LVZs on voltage maps (14, 24). In addition, LA enlargement has been closely associated with LVZ development (24), as both increased LA volume and chronic atrial stretch are key contributors to atrial substrate remodeling (25). Multiple studies have also highlighted an association between female sex and the presence of LVZs (25, 26). Women typically develop AF later in life, often after menopause, suggesting that hormonal changes combined with cardiovascular risk factors may contribute to the progression of LA fibrosis in post-menopausal women, although this mechanism remains to be fully elucidated (27). In our cohort of patients with persistent AF, we further observed that PWD ≥150 ms was predictive of LVZs. This finding is consistent with results from Jadidi et al., who demonstrated that PWD ≥150 ms identifies patients with advanced LA LVZ and a high risk of arrhythmia recurrence following PVI alone (23).

Interestingly, in our cohort, DAT was not identified as an independent predictor of LVZ occurrence. Furthermore, after applying IPTW, the previously observed difference in LVZ prevalence was no longer significant. The relatively small sample size may have limited the statistical power needed to detect significant differences. Nevertheless, the use of IPTW was intended to minimize baseline differences and isolate the effect of DAT from confounding cardiovascular risk factors. These findings suggest that DAT alone may not be sufficient to predict LVZ development or the degree of atrial remodeling. Other factors such as age, female sex, or lifestyle-related factors (obesity, smoking, alcohol excess, hypertension, and OSA) are likely to play critical roles in LA remodeling. It is now well established that LA remodeling can precede the onset of AF (8).

In our study, DAT was defined as the time between the first documented AF episode and the ablation date. This definition may underestimate the true AF duration, particularly in asymptomatic patients, and may reflect health care access rather than disease biology. Nevertheless, a 1-year cutoff was used to align with prior studies (10) and with common clinical practice, in which ablation is often considered within 6–12 months after diagnosis. To avoid potential bias from dichotomization, sensitivity analyses treating DAT as a continuous variable (OR per 30-day increase) and using restricted cubic splines were performed. These analyses did not demonstrate a significant association or non-linear relationship between DAT and LVZ, supporting the robustness of our findings.

A history of paroxysmal AF was, however, associated with a lower probability of LVZs. This finding may reflect a slower progression of atrial cardiomyopathy rather than a direct protective effect. In patients progressing from paroxysmal to persistent AF, atrial remodeling may develop more slowly, likely because prior use of antiarrhythmic drugs reduces the AF burden. By contrast, some patients with previously silent paroxysmal AF had not received therapy to limit this burden, which likely promoted faster atrial remodeling and progression to symptomatic persistent AF.

Walters et al. (21) demonstrated that in patients with paroxysmal AF, a high AF burden (≥10%) promotes progressive atrial remodeling, whereas a low burden tends to remain stable.

From a pathophysiological perspective, persistent AF following a paroxysmal phase may reflect a slower, progressive evolution, with a predominance of trigger-related mechanisms and mainly electrical remodeling. Finally, patients presenting directly with persistent AF, without a documented paroxysmal history, may already exhibit substantial pre-existing left atrial remodeling, independent of any prior paroxysmal phase.

These observations support the notion that the influence of DAT alone on atrial remodeling may be less significant than that of cumulative risk factors. Therefore, the traditional concept of “AF begets AF,” first proposed in 1995 to describe how AF promotes its own perpetuation through progressive atrial remodeling (initially electrical, then structural) (28, 29), may not fully capture the complexity of atrial substrate changes.

More recent work by Bergonti et al. has shown that areas of moderately decreased voltage, intermediate between healthy and fibrotic tissue, are often part of a continuous spectrum of atrial remodeling rather than a simple binary classification. They also observed that LAVI generally correlates with LVZs. However, a subset of patients in their study exhibited LVZs despite a normal LAVI, highlighting the multifactorial nature of atrial remodeling (30).

The recently introduced concept of atrial cardiomyopathy (AtCM) offers a potential explanation for atrial remodeling that precedes AF. The AtCM was the subject of a consensus article that defined it as the set of functional and structural changes, also called remodeling, of the atrial myocardium. In this framework, AF may represent a contributing or aggravating factor rather than the primary driver of atrial remodeling (31). All of these findings underscore the multifactorial origins of LVZ development and the complex interplay between LA remodeling and enlargement, AF burden, and various underlying risk factors.

### LVZ-guided ablation results and predictors of AT recurrence

Increasing evidence supports the association between ERC therapy and a lower risk of adverse cardiovascular outcomes (3). Among existing ERC strategies, the AF ablation procedure was more effective than AAD therapy in maintaining SR (32). In our analysis of a cohort of patients with persistent AF, DAT ≤1 year is an important predictor of CA outcomes.

Indeed, the patients of our cohort undergoing ablation beyond the first year after AF diagnosis experienced a lower AT recurrence with more AAD discontinuation than the DAT >1 year group. These findings are in line with previous reports. Hussein et al. demonstrated that first-quartile (DAT ≤1 year) patients had significantly fewer recurrences than those in quartiles 2–4 in persistent AF (10). De Greef et al. reported results comparable to ours, noting that early PVI timing is associated with better long-term outcomes, with the optimal timing within the first year of diagnosis (9). Bisbal et al. observed that patients undergoing ablation beyond the first year from AF diagnosis had a 4-fold increased risk of recurrent AF and repeat ablation (33). Our study, in addition to the previous studies mentioned above, supported the 1-year DAT cutoff, which was further reinforced by the study of De Greef et al. (34). They analyzed the impact of DAT on 3-year AF recurrence in 2,000 patients undergoing PVI. In their analysis, an immediate steep rise from 27% to 40% was observed in the first 36 months, after which the impact of DAT becomes less pronounced and almost insignificant beyond 90 months (34).

Our study also investigated the efficacy of voltage-guided ablation in patients with persistent AF stratified by DAT. Currently, limited information is available on the efficacy and safety of voltage-guided ablation in patients with AF stratified by DAT. Our study is among the first to assess outcomes of LVZ-guided RF ablation in patients with persistent AF in relation to DAT. Hussein et al. reported that arrhythmia recurrence rates over 2 years were 33.6% and 52.6% in patients with DAT ≤1 and 1.1–3.0 years, respectively (10). Similarly, Bisbal et al. reported AF recurrence rates of 33% and 50%–55% at 2 years in patients with persistent AF and DAT≤1 year and DAT>1 year, respectively (33). Hussein et al. performed ablation of complex fractionated atrial electrograms, with or without superior vena cava ablation, after PVI at the discretion of the ablating physicians (10). For Bisbal et al., additional ablation lines or ablation of complex fractionated electrograms were performed in addition to PVI according to the protocol of each hospital (33).

In our cohort of patients with persistent AF, we observed better outcomes: AF recurrence rates of 15% vs. 26% at 2 years in patients with DAT≤1 year and DAT>1 year, respectively. Notably, 75% of patients were able to discontinue AADs. PVI alone was performed in 72% of patients with a shorter DAT, compared with 57% in patients with a longer DAT, thereby limiting substrate ablation and LA scar formation.

A recent multicenter randomized trial demonstrated that LVZ-guided ablation in addition to PVI was superior to PVI alone in patients with persistent AF (35). Further randomized studies are needed to validate these findings.

In several studies, DAT was the strongest predictor of arrhythmia recurrence in large cohorts (9, 10, 33). Similarly, a shorter duration (<1 year) between AF diagnosis and ablation has been identified as a key protective factor against AF recurrence after CA (36).

We could not identify any significant predictors of AT recurrence after CA in our cohort. This lack of significant findings may be attributed to the small sample size, which restricted the statistical power to detect meaningful associations. The strategy of ablation may also explain the absence of identification of predictive factors of recurrence after CA.

### Study limitations

Our study was a single-center, non-randomized, observational study with a retrospective design, and the limited number of patients may have affected the results. The clinical course between AF diagnosis and CA was not detailed. Unmeasured factors (antiarrhythmic drug trials, cardioversion frequency, and risk-factor modifications) may have influenced atrial remodeling and post-ablation outcomes, introducing residual confounding. The CA results were assessed for up to 36 months post-ablation. A longer follow-up could be of interest, especially for the repeat-procedure assessment. In addition, the discontinuation of AADs could not be achieved for the whole cohort because the follow-up was performed by the individual cardiologists of patients, which could have influenced the results of ablation. Recurrence detection was based on scheduled 12-lead ECGs and 24-h Holter monitoring, supplemented by symptom-triggered ECG recordings; this approach may have missed asymptomatic or intermittent episodes. Further multicenter randomized studies are warranted to assess long-term outcomes after voltage-guided AF ablation according to DAT.

## Conclusion

DAT >1 year was associated with more advanced LA electrophysiological remodeling characterized by lower bipolar voltage, a greater extent of LVZ, and increased LA volume. During the 36-month follow-up after a single voltage-guided ablation, patients with a longer DAT experienced a higher rate of AT recurrence. Female sex, advanced age, indexed LA volume, and PWD ≥150 ms were identified as independent predictors of LVZs across the cohort. While DAT alone may not fully account for the complex mechanisms underlying atrial remodeling, it clearly plays a significant role in patient prognosis. These findings support the promotion of early AF ablation, ideally within the first year following diagnosis, to help limit the progression of structural and electrical LA remodeling.

## Data Availability

The original contributions presented in the study are included in the article/Supplementary Material, further inquiries can be directed to the corresponding author.

## References

[1] Benjamin EJ Wolf PA D’Agostino RB Silbershatz H Kannel WB Levy D. Impact of atrial fibrillation on the risk of death: the Framingham Heart Study. Circulation. (1998) 98(10):946–52. 10.1161/01.CIR.98.10.946 9737513

[2] Wolf PA Dawber TR Thomas HE Jr Kannel WB. Epidemiologic assessment of chronic atrial fibrillation and risk of stroke: the Framingham study. Neurology. (1978) 28(10):973–7. 10.1212/WNL.28.10.973 570666

[3] Kirchhof P Camm AJ Goette A Brandes A Eckardt L Elvan A Early rhythm- control therapy in patients with atrial fibrillation. N Engl J Med. (2020) 383(14):1305–16. 10.1056/NEJMoa2019422 32865375

[4] Proietti M Vitolo M Harrison SL Lane DA Fauchier L Marin F Real-world applicability and impact of early rhythm control for European patients with atrial fibrillation: a report from the ESC-EHRA EORP-AF long-term general registry. Clin Res Cardiol. (2022) 111:70–84. 10.1007/s00392-021-01914-y 34448931 PMC8766399

[5] Seki Y Fujisawa T Ikemura N Ibe S Tsuzuki I Hashimoto K Catheter ablation improves outcomes and quality of life in Japanese patients with early-stage atrial fibrillation: a retrospective cohort study. Heart Rhythm. (2022) 19(7):1076–83. 10.1016/j.hrthm.2022.02.017 35183738

[6] Andrade JG Wazni OM Kuniss M Hawkins NM Deyell MW Chierchia GB Cryoballoon ablation as initial treatment for atrial fibrillation. J Am Coll Cardiol. (2021) 78(9):914–30. 10.1016/j.jacc.2021.06.038 34446164

[7] Andrade JG Deyell MW Macle L Wells GA Bennett M Essebag V Progression of atrial fibrillation after cryoballoon or drug therapy. N Engl J Med. (2023) 388(2):105–16. 10.1056/NEJMoa2212540 36342178

[8] Hindricks G Potpara T Dagres N Arbelo E Bax JJ Blomström-Lundqvist C 2020 ESC guidelines for the diagnosis and management of atrial fibrillation developed in collaboration with the European Association for Cardio-Thoracic Surgery (EACTS): the task force for the diagnosis and management of atrial fibrillation of the European Society of Cardiology (ESC) developed with the special contribution of the European Heart Rhythm Association (EHRA) of the ESC. Eur Heart J. (2020) 42:373–498. 10.1093/eurheartj/ehaa612 32860505

[9] De Greef Y Schwagten B Chierchia GB de Asmundis C Stockman D Buysschaert I. Diagnosis-to-ablation time as a predictor of success: early choice for pulmonary vein isolation and long-term outcome in atrial fibrillation: results from the Middelheim-PVI registry. EP Europace. (2018) 20(4):589–95. 10.1093/europace/euw426 28340103

[10] Hussein AA Saliba WI Barakat A Bassiouny M Chamsi-Pasha M Al-Bawardy R Radiofrequency ablation of persistent atrial fibrillation: diagnosis-to-ablation time, markers of pathways of atrial remodeling, and outcomes. Circul Arrhythmia Electrophysiol. (2016) 9(1):e003669. 10.1161/CIRCEP.115.003669 PMC5349298 26763227

[11] Winkle RA Mead RH Engel G Salcedo J Brodt C Barberini P Early ablation of newly diagnosed paroxysmal atrial fibrillation (NEWPaAF) versus newly diagnosed persistent atrial fibrillation (NEWPeAF): comparison of patient populations and ablation outcomes. J Cardiovasc Electrophysiol. (2024) 35:984–93. 10.1111/jce.16248 38486082

[12] Yamaguchi T Tsuchiya T Nagamoto Y Miyamoto K Murotani K Okishige K Long-term of pulmonary vein antrum isolation in patients with atrial fibrillation: an analysis in regards to substrates and pulmonary vein reconnections. Europace. (2014) 16(4):511–20. 10.1093/europace/eut265 24078342

[13] Huo Y Gaspar T Pohl M Sitzy J Richter U Neudeck S Prevalence and predictors of low voltage zones in the left atrium in patients with atrial fibrillation. Europace. (2018) 20(6):956–62. 10.1093/europace/eux082 28605524

[14] Teh AW Kistler PM Lee G Medi C Heck PM Spence SJ Long-term effects of catheter ablation for lone atrial fibrillation: progressive atrial electroanatomic substrate remodeling despite successful ablation. Heart Rhythm. (2012) 9(4):473–80. 10.1016/j.hrthm.2011.11.013 22079885

[15] Mahnkopf C Badger TJ Burgon NS Daccarett M Haslam TS Badger CT Evaluation of the left atrial substrate in patients with lone atrial fibrillation using delayed-enhanced MRI: implications for disease progression and response to catheter ablation. Heart Rhythm. (2010) 7:1475–81. 10.1016/j.hrthm.2010.06.030 20601148 PMC3106345

[16] Phlips T Taghji P El Haddad M Wolf M Knecht S Vandekerckhove Y Improving procedural and one-year outcome after contact force-guided pulmonary vein isolation: the role of interlesion distance, ablation index, and contact force variability in the “CLOSE”-protocol. Europace. (2018) 20:f419–27. 10.1093/europace/eux376 29315411

[17] Okamatsu H Koyama J Sakai Y Negishi K Hayashi K Tsurugi T High-power application is associated with shorter procedure time and higher rate of first-pass pulmonary vein isolation in ablation index-guided atrial fibrillation ablation. J Cardiovasc Electrophysiol. (2019) 30:2751–8. 10.1111/jce.14223 31600006

[18] Austin PC Stuart EA. Moving towards best practice when using inverse probability of treatment weighting (IPTW) using the propensity score to estimate causal treatment effects in observational studies. Stat Med. (2015) 34(28):3661–79. 10.1002/sim.6607 26238958 PMC4626409

[19] De Vos CB Pisters R Nieuwlaat R Prins MH Tieleman RG Coelen RJS Progression from paroxysmal to persistent atrial fibrillation. J Am Coll Cardiol. (2010) 55(8):725–31. 10.1016/j.jacc.2009.11.040 20170808

[20] Lang RM Badano LP Mor-Avi V Afilalo J Armstrong A Ernande L Recommendations for cardiac chamber quantification by echocardiography in adults: an update from the American Society of Echocardiography and the European Association of Cardiovascular Imaging. J Am Soc Echocardiography. (2015) 28(1):1–39.e14. 10.1016/j.echo.2014.10.003 25559473

[21] Walters TE Nisbet A Morris GM Tan G Mearns M Teo E Progression of atrial remodeling in patients with high-burden atrial fibrillation: implications for early ablative intervention. Heart Rhythm. (2016) 13:331–9. 10.1016/j.hrthm.2015.10.028 26484789

[22] Marzak H Rivière H Fitouchi S Cardi T Kanso M Morel O The influence of left atrial volume on left atrial voltage in persistent atrial fibrillation patients without low-voltage zone: outcomes of pulmonary vein isolation. Europace. (2024) 26(7):euae190. 10.1093/europace/euae190 39031019 PMC11259848

[23] Jadidi A Müller-Edenborn B Chen J Keyl C Weber R Allgeieret J The duration of the amplified sinus-P-wave identifies presence of left atrial low voltage substrate and predicts outcome after pulmonary vein isolation in patients with persistent atrial fibrillation. JACC Clin Electrophysiol. (2018) 4(4):531–43. 10.1016/j.jacep.2017.12.001 30067494

[24] Ammar-Busch S Buiatti A Tatzber A Reents T Bourier F Semmler V Predictors of low voltage areas in persistent atrial fibrillation: is it really a matter of time? J Interv Card Electrophysiol. (2020) 57(3):345–52. 10.1007/s10840-018-0471-7 30374659

[25] Wang XH Li Z Mao JL Zang MH Pu J. Low voltage areas in paroxysmal atrial fibrillation: the prevalence, risk factors and impact on the effectiveness of catheter ablation. Int J Cardiol. (2018) 269:139–44. 10.1016/j.ijcard.2018.07.076 30060968

[26] Marzak H Ringele R Matsushita K Marchandot B Fitouchi S Cardi T Impact of gender on left atrial low-voltage zones in patients with persistent atrial fibrillation: results of voltage-guided ablation. Front Cardiovasc Med. (2023) 10:1229345. 10.3389/fcvm.2023.1229345 37692044 PMC10484507

[27] Rosano GM Leonardo F Dicandia C Sheiban I Pagnotta P Pappone C Acute electrophysiologic effect of oestradiol 17beta in menopausal women. Am J Cardiol. (2000) 86(12):1385–7. 10.1016/S0002-9149(00)01251-0 11113421

[28] Wijffels MC Kirchhof CJ Dorland R Allessie MA. Atrial fibrillation begets atrial fibrillation. A study in awake chronically instrumented goats. Circulation. (1995) 92:1954–68. 10.1161/01.CIR.92.7.1954 7671380

[29] Kamalvand K Tan K Lloyd G Gill J Bucknall C Sulke N. Alterations in atrial electrophysiology associated with chronic atrial fibrillation in man. Eur Heart J. (1999) 20:888–95. 10.1053/euhj.1998.1404 10329094

[30] Bergonti M Spera FR Ferrero TG Nsahlai M Bonomi A Tijskens M Characterization of atrial substrate to predict the success of pulmonary vein isolation: the prospective, multicenter MASH-AF II (multipolar atrial substrate high density mapping in atrial fibrillation) study. J Am Heart Assoc. (2023) 12:e027795. 10.1161/JAHA.122.027795 36565183 PMC9973584

[31] Goette A DCorradi D Dobrev D Aguinaga L Cabrera JA Chugh SS Atrial Cardiomyopathy Revisited—Evolution of a Concept. A Clinical Consensus Statement of the European Heart Rhythm Association (EHRA) of the ESC, the Heart Rhythm Society (HRS), the Asian Pacific Heart Rhythm Association (APHRS), and the Latin American Heart Rhythm Society (LAHRS). Europace. (2024) 26(9):euae204. 10.1093/europace/euae204 39077825 PMC11431804

[32] Packer DL Mark DB Robb RA Monahan KH Bahnson TD Poole JE Effect of catheter ablation vs antiarrhythmic drug therapy on mortality, stroke, bleeding, and cardiac arrest among patients with atrial fibrillation: the CABANA randomized clinical trial. JAMA. (2019) 321(13):1261–74. 10.1001/jama.2019.0693 30874766 PMC6450284

[33] Bisbal F Alarcón F Ferrero-De-Loma-Osorio A Gonzalez-Ferrer JJ Alonso-Martin C Pachon M Diagnosis-to-ablation time in atrial fibrillation: a modifiable factor relevant to clinical outcome. J Cardiovasc Electrophysiol. (2019) 30(9):1483–90. 10.1111/jce.14000 31115940

[34] De Greef Y Bogaerts K Sofianos D Buysschaert I. Impact of diagnosis-to-ablation time on AF recurrence: pronounced the first 3 years, irrelevant thereafter. JACC Clin Electrophysiol. (2023) 9(11):2263–72. 10.1016/j.jacep.2023.07.008 37656100

[35] Huo Y Gaspar T Schönbauer R Wójcik M Fiedler L Roithinger FX Low-voltage myocardium-guided ablation trial of persistent atrial fibrillation. NEJM Evid. (2022) 1(10):EVIDoa2200141. 10.1056/EVIDoa2200141 38319851

[36] Charitakis E Dragioti E Stratinaki M Korela D Tzeis S Almroth H Predictors of recurrence after catheter ablation and electrical cardioversion of atrial fibrillation: an umbrella review of meta-analyses. Europace. (2023) 25(1):40–8. 10.1093/europace/euac143 36037026 PMC10103559

