# Supplementary material for: Impact of diagnosis-to-ablation time on left atrial remodeling and voltage-guided ablation outcome in persistent atrial fibrillation patients
Source: Front Cardiovasc Med. 2026 Mar 12;13:1751432. doi: 10.3389/fcvm.2026.1751432 (PMC13017272; doi:10.3389/fcvm.2026.1751432)
Supplement: Supplementary file 1 [file Datasheet1.doc]

**Table S1.** Procedural data related characteristics according to DAT after IPTW.

| **Variable** | **DAT ≤ 1 year**  **(n = 129.7)** | **DAT > 1 year**  **(n = 218.7)** | **RR [95%CI]** | **P value** |
| --- | --- | --- | --- | --- |
| Only PVI, n (%) | 91.4 (70.4) | 125.2 (57.4) | 0.57 [0.30 - 1.06] | 0.076 |
| LVZ ablation, n (%) | 38.3 (29.6) | 92.8 (42.6) | 1.77 [0.94 - 3.30] | 0.076 |
| Complications, n (%) | 7.4 (5.7) | 7.6 (3.5) | 0.60 [0.18 - 1.99] | 0.405 |
| Scarpa’s hematoma, n (%) | 5.4 (4.1) | 3.3 (1.5) | 0.35 [0.07 - 1.79] | 0.199 |

All data are presented as a value (percentage) for categorical variables or median (25^th^-75^th^ percentile) for quantitative variables. A two-tailed p value<0.05 was considered significant.

**Abbreviations:** *IPTW, inverse probability of treatment weighting ; LVZ, Low voltage zone; RR, Relative risk; CI, confidence interval; DAT, diagnosis‐to‐ablation time ; PVI, pulmonary vein isolation.*

**Table S2.** Multivariable analysis of predictors of Low-Voltage Zones, including DAT as a continuous variable.

|  | **Multivariable Analysis** | | |
| --- | --- | --- | --- |
|  | OR | 95% CI | P Value |
| DAT * | 1.00 | [0.99 - 1.01] | 0.697 |
| Age 60-75 years | 4.32 | [1.66 - 11.28] | 0.003 |
| Age > 75 years | 5.69 | [1.49 - 21.66] | 0.011 |
| Female gender | 4.85 | [2.41 - 9.76] | <0.001 |
| Hypertension | 1.53 | [0.69 - 3.40] | 0.295 |
| Diabetes mellitus | 1.66 | [0.72 - 3.84] | 0.235 |
| CHA_2_DS_2_-VASc score ≥2 | 1.78 | [0.67 - 4.75] | 0.247 |
| eGFR < 60 ml/ min/1,73² | 0.95 | [0.40 - 2.26] | 0.911 |
| LAVI on CT ≥ 48 ml.m^2^ | 11.79 | [2.16 - 64.43] | 0.004 |
| PWD ≥ 150 ms | 11.18 | [5.79 - 21.58] | <0.001 |
| Paroxysmal AF history | 0.51 | [0.26 - 1.01] | 0.054 |

Data are presented as an odd ratio with 95% CI. *A two-tailed p value<0.05 was considered significant*.

**Abbreviations:** *OR, odds ratio; CI, confidence interval; DAT, diagnosis‐to‐ablation time;* *eGFR, estimated glomerular filtration rate; AF, atrial fibrillation; LAVI, left atrial volume index; CT, computed tomography; PWD, P-wave duration.*

Diagnosis‐to‐ablation time = time from first clinical diagnosis of AF to ablation procedure.

*OR associated with a 30-day increase in delay.

**Figure S1.** Mean absolute standardized differences (ASD) of the different variables before and after weighting on the two groups of DAT.


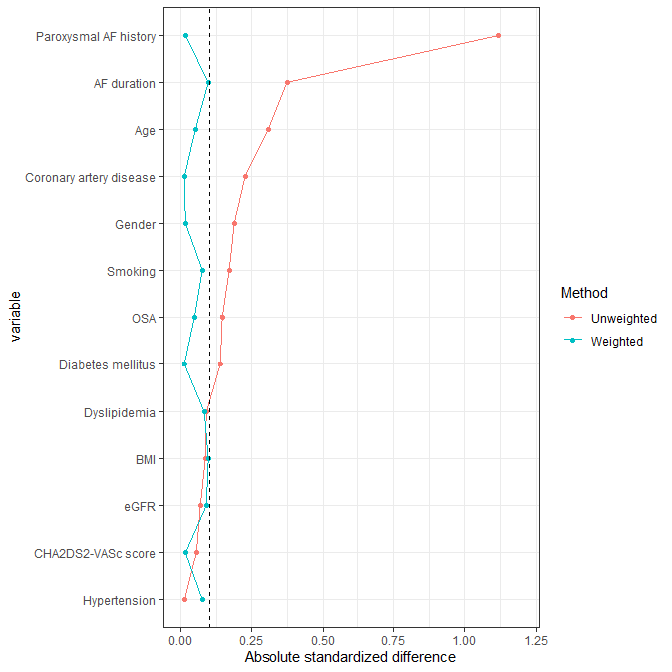


Absolute standardized differences (ASD) were used to determine the influence of covariates on the two groups. Groups are generally considered similar when the ASD is < 0,1.

**Abbreviations:** *AF, atriale fibrillation ;* *ASD, absolute standardized differences ; OSA, obstructive sleep apnea ; BMI, body mass index ; eGFR, estimated glomerular filtration rate; DAT, diagnosis‐to‐ablation time.*

**Figure S2.** Restricted Cubic Spline Analysis of the Association Between DAT and Low-Voltage Zones.


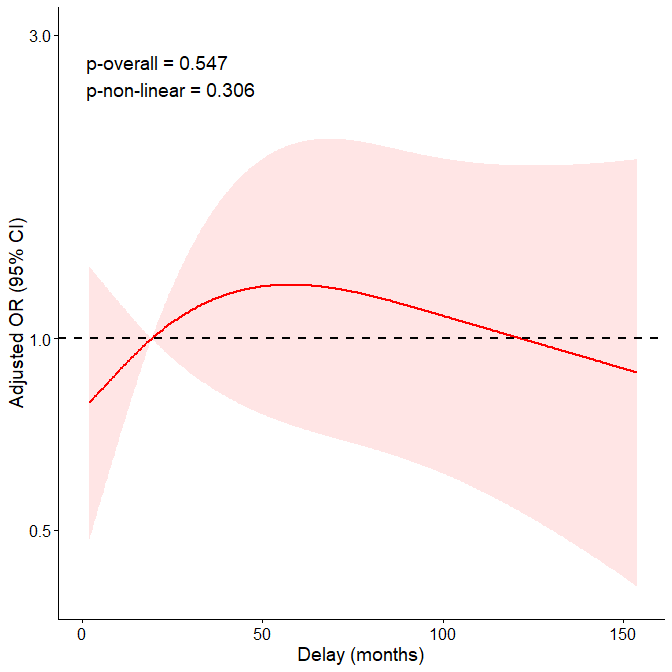


Data are presented as an odd ratio with 95% CI. *A two-tailed p value<0.05 was considered significant*.

**Abbreviations:** *OR, odds ratio; DAT, diagnosis‐to‐ablation time.*

Diagnosis‐to‐ablation time = time from first clinical diagnosis of AF to ablation procedure.
